# Waste-Towel-Derived Hard Carbon as High Performance Anode for Sodium Ion Battery

**DOI:** 10.3390/polym18020206

**Published:** 2026-01-12

**Authors:** Daofa Ying, Kuo Chen, Jiarui Liu, Ziqian Xiang, Jiazheng Lu, Chuanping Wu, Baohui Chen, Yang Lyu, Yutao Liu, Zhen Fang

**Affiliations:** State Key Laboratory of Disaster Prevention and Reduction for Power Grid Transmission and Distribution Equipment, State Grid Hunan Electric Company Limited Disaster Prevention and Reduction Center, Changsha, 410100, China

**Keywords:** waste towel, hard carbon, sodium ion battery

## Abstract

Developing cost-effective yet high-performance hard carbon anodes is critical for advancing the commercialization of sodium-ion batteries (SIBs), as they offer a balance of low cost, high capacity, and compatibility with Na^+^ storage mechanisms. Herein, waste towels, an abundant, low-cost precursor with a high carbon yield (>49%), were utilized to synthesize hard carbons via a two-step process: pre-oxidation at 250 °C to stabilize the fibrous structure, followed by carbonization at 1100 °C (THC-1100), 1300 °C (THC-1300), or 1500 °C (THC-1500). Electrochemical evaluations revealed that THC-1300, carbonized at an intermediate temperature, exhibited superior Na^+^ storage performance compared to its counterparts: it delivered a high reversible specific capacity of ~320 mAh/g at 1.0 C (1 C = 320 mA/g), with 78% capacity retention after 200 cycles, demonstrating excellent long-term cyclic stability. Its rate capability was equally impressive, achieving specific capacities of 341.5, 331.2, 302.0 and 234.8 mAh/g at 0.2, 0.5, 2.0 and 5.0 C, respectively, indicating efficient Na^+^ diffusion even at high current densities. Notably, THC-1300 also showed an improved initial Coulombic efficiency (ICE) of 75.4%, reflecting reduced irreversible Na^+^ consumption during the first cycle. These enhancements are attributed to the synergistic effects of THC-1300’s optimized structural and textural properties: a balanced interlayer spacing (d_(002)_ = 0.387 nm) that facilitates rapid Na^+^ intercalation, a low BET surface area (1.62 m^2^/g) helps to minimize electrolyte side reactions. The combined advantages of high specific capacity, improved ICE, and remarkable cycling stability position this waste-towel-derived hard carbon as a highly viable and sustainable candidate for anode materials in next-generation SIBs, addressing both performance and cost requirements for large-scale energy storage applications.

## 1. Introduction

The rapid expansion of renewable energy systems, electric vehicles, and portable electronics has generated substantial demand for advanced energy storage technologies [1]. Currently, this growing energy demand is largely met by lithium-ion batteries (LIBs), which dominate the markets for (hybrid) electric vehicles and portable electronics [2]. Sodium-ion batteries (SIBs) have attracted considerable research interest as a promising alternative due to the natural abundance of sodium, leading to potential cost-effectiveness and enhanced safety [3,4]. While significant progress has been made in developing promising cathode materials for SIBs, a considerable challenge remains in identifying suitable anode materials. Existing anode materials can be categorized into insertion [5], conversion [6,7], and alloy-types [8] based on their sodium storage mechanisms. Although conversion and alloy-type anodes exhibit high specific capacities enabled by multi-electron reactions, they often suffer from poor structural stability caused by severe volume expansion during cycling [9,10]. In contrast, insertion-type anodes typically demonstrate superior structural stability, as they accommodate ions through a simple insertion/de-insertion process with minimal volume change.

Graphite, a classic carbon-based insertion anode, performs excellently in LIBs by forming a stable LiC_6_ intercalation compound [11]. However, it is considered unsuitable for SIBs due to the thermodynamic instability of the corresponding NaC_6_ compound. Meanwhile, other carbon materials such as carbon dots [12], carbon nanofiber [13] and their derivatives have also been employed as promising anode materials due to their unique structures. However, these materials face commercialization barriers owing to their high-surface-area-induced poor initial efficiency, unstable cycling performance, and uncompetitive costs. Consequently, the development of non-graphitic carbon materials is essential for the commercialization of SIBs. Among these, hard carbon is a kind of non-graphitizable and highly disordered carbon—has emerged as a leading anode candidate [14], owing to its low operating potential plateau (<0.2 V) and respectable specific capacity (>300 mAh/g) [15]. Hard carbon can be synthesized from various low-cost, carbon-rich precursors. To further reduce the cost and environmental footprint of SIBs, recent research has focused on deriving hard carbons from sustainable biomass resources [1,16]. Waste biomass sources such as rice husk [17], banana peel [18], and peanut shells [19,20] have been explored, as they can produce hard carbons with unique microstructures, cost-effectiveness and eco-friendliness [21].

However, biomass-derived hard carbons are confronted with multiple challenges that hinder their widespread adoption [1]. A primary concern is their generally low Initial Coulombic Efficiency (ICE), which is critical for the energy density of a full cell [22]. For instance, hard carbons derived from rice husk and kelp have reported ICEs of only 64% [17] and 56%, respectively. Furthermore, issues such as low production yield and poor batch-to-batch consistency also impede their scalable manufacturing. Research indicates that properties like ICE can be improved by minimizing structural defects and reducing the specific surface area [23]. These characteristics can be effectively tailored through simple process parameters, such as the pyrolysis temperature.

In this work, we report the synthesis of high-performance hard carbon anodes from a low-cost and abundant waste precursor: discarded towels. To the best of our knowledge, this specific household waste has not been previously investigated for SIB anodes. We systematically studied the effect of pyrolysis temperature (1100, 1300, and 1500 °C) on the microstructure and electrochemical performance. The hard carbon synthesized at 1300 °C demonstrated a high reversible capacity, exceptional cycling stability, and most notably, a significantly improved ICE compared to many other biomass-derived carbons. This study demonstrates that utilizing waste towels as a precursor, combined with optimized pyrolysis conditions, presents a feasible strategy to produce cost-effective and high-performance hard carbon anodes for practical sodium-ion storage.

## 2. Experimental Sections

### 2.1. Materials and Methods

**Figure 1:**
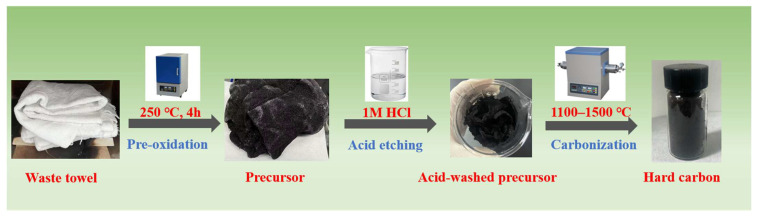
Schematic diagram of experimental process.

The preparation process of hard carbon from waste towels involves three key steps (Figure 1): First, waste towels are subjected to pre-oxidation in a muffle furnace at 250 °C for 4 h under air atmosphere to stabilize the cellulose structure, resulting in a dark brown brittle precursor. Next, the pre-oxidized precursor is ground and immersed in 1 M HCl solution at 80 °C for 12 h for acid etching, followed by filtration, neutralization washing, and drying to obtain the acid-washed precursor with reduced impurities and enhanced surface roughness. Finally, the purified precursor is carbonized in a tubular furnace under argon atmosphere at 1100–1500 °C for 2 h, leading to the formation of hard carbon with a disordered turbostratic structure, optimal interlayer spacing (0.37–0.40 nm), and hierarchical porous morphology suitable for sodium-ion storage. The obtained hard caron materials from different temperature is designated as THC-1100, THC-1300 and THC-1500.

### 2.2. Structural, Surface and Morphological Characterization

Powder XRD analysis was performed using X-ray diffraction (Rigaku Miniflex600, Rigaku Corporation, Tokyo, Japan) with Cu@Ka radiation at 5°/min. The inter- layer spacing (d002) is calculated using the hard carbon characteristic peak (002) and Bragg’s Equation (1).

(1) $$d_{\left(002\right)}=\frac{\;\lambda }{2\sin\theta }$$

λ is wavelength of X-ray radiation (0.15406 nm), and θ is Bragg’s angle in degree.

Raman spectra are acquired with the help of alpha 300 RAS WiTec spectrometer UHTS300 (WITec, Ulm, Germany) attached with LASER of 532 nm wavelength in range of 1000–2000 cm^−1^. The Fourier transform infrared (FT-IR) spectra of the THC samples were recorded using a Thermo Scientific K-Alpha spectrometer (Thermo Fisher Scientific, Waltham, MA, USA) equipped with a single-reflection diamond ATR (Attenuated Total Reflectance) accessory. Spectral range: 4000–400 cm^−1^. Nitrogen adsorption–desorption measurements were conducted with degassing at 150 °C for 12 h (full pore mode), and pore size distribution was derived from the desorption branch using the BJH method. Morphology information was obtained via scanning electron microscopy (Zeiss, SIG-MA, Carl Zeiss AG, Oberkochen, Germany). The transmission electron microscope (TEM) images and SAED patterns, etc., were obtained using a transmission electron microscope (Tecnai, FEI Company, Tokyo, Japan, 200 kV).

### 2.3. Electrochemical Characterization

Cyclic voltammetry (CV) of the button cells assembled with different samples were tested by CHI660f (Shanghai Chenhua Instrument, Shanghai, China) electrochemical workstation.

Coin Cell Evaluation: The coin cells was fabricated by homogenizing active material, acetylene black, and polyvinylidene fluoride (PVDF) in an 8:1:1 weight ratio within N-methyl-2-pyrrolidone (NMP) solvent. The slurry was blade-coated onto 15 μm double-sided carbon-coated aluminum foil (1 μm carbon layer per side), yielding an active material areal loading of 6 mg/cm^2^ and electrode thickness of 70 ± 1 μm. CR2032 coin cells were assembled in an argon-filled glovebox (<0.1 ppm O_2_/H_2_O) using sodium foil counter electrodes, a 16 mm diameter separator, and 1 M NaPF_6_ in DME/DOL (5:1 *v*/*v*) electrolyte. Cycling performance was evaluated via constant current charge–discharge tests (LAND CT3002A testing system, 0 to 2.0 V). C-rate capability was assessed by cycling at discharge currents of 0.2, 0.5, 1.0, 2.0 and 5.0 C.

## 3. Results and Discussion

**Figure 2:**
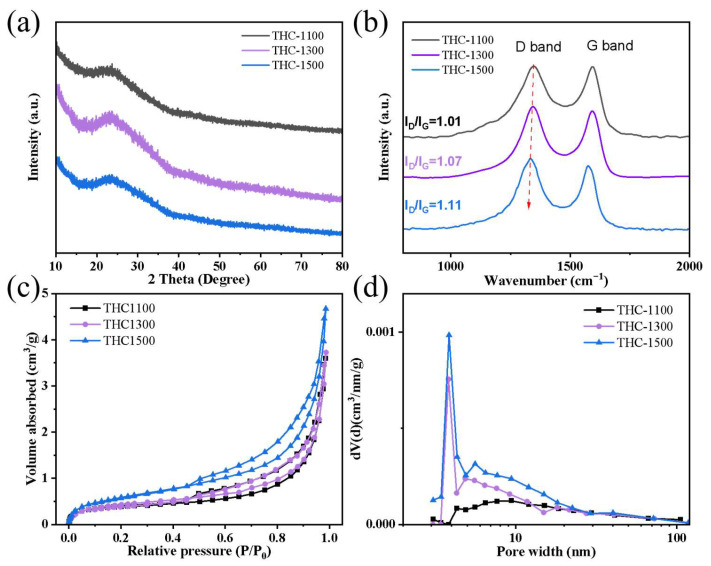
(**a**) Powder XRD pattern, (**b**) Raman spectra, (**c**) N_2_ adsorption/desorption isotherms and (**d**) BJH pore size distribution of THC-1000, THC-1300 and HC1500.

A high hard carbon yield (>49%) was achieved via a pre-oxidation strategy. Notably, the yield decreased only slightly with increasing carbonization temperature, THC-1500 still retained a yield of 49.3%. The structural properties of hard carbons (THCs) derived from waste towels are systematically tailored by carbonization temperature (1100–1500 °C), as revealed in Figure 2. X-ray diffraction (XRD) patterns (Figure 2a) show two broad characteristic peaks centered at ~23° and ~43°, corresponding to the (002) and (100) planes of turbostratic carbon, respectively. With increasing carbonization temperature from 1100 °C to 1500 °C, the (002) peak shifts slightly toward higher 2θ angles (from 23.1° to 23.5°), indicating a reduction in interlayer spacing d_(002)_ from 0.385 nm to 0.378 nm (calculated via Bragg’s law). This narrowing of d_(002)_ is attributed to the enhanced ordering of graphitic microdomains at higher temperatures [24], consistent with the gradual elimination of lattice defects and the growth of sp^2^-hybridized carbon layers. Notably, all d_(002)_ values fall within the optimal range (0.37–0.40 nm) for sodium-ion storage, balancing interlayer Na^+^ insertion (platform capacity) and surface adsorption (slope capacity) [21,25]. Raman spectroscopy (Figure 2b) further confirms the structural evolution, with two prominent peaks at ~1350 cm^−1^ (D-band, disordered carbon) and ~1580 cm^−1^ (G-band, graphitic carbon) [26] The intensity ratio I_D_/I_G_ increases from 1.01 (THC-1100) to 1.11 (THC-1500), the rising I_D_/I_G_ ratio primarily reflects an increase in edge/planar defects within the turbostratic graphitic domains, rather than overall amorphization. Nitrogen adsorption–desorption isotherms (Figure 2c) and pore size distributions (PSDs, Figure 2d) highlight the textural evolution. All samples exhibit type IV isotherms with H4 hysteresis loops, characteristic of hierarchical micro-mesoporous structures. The structural evolution induced by carbonization temperature critically governs the textural properties of the prepared hard carbons. Specifically, the specific surface area (SSA) increases from 1.44 m^2^/g (THC-1100) to 2.22 m^2^/g (THC-1500), while the total pore volume similarly rises from 0.0057 cm^3^/g to 0.0073 cm^3^/g. This variation may attributed to the progressive development of pore structure during high-temperature treatment, where the release of volatile species creates new micropores while partial pore coalescence occurs simultaneously. Overall, the THC exhibits a dense structure with low porosity, which can minimize side reactions with the electrolyte. Pore size distribution (PSD) analysis reveals a dominant micropore population (<2 nm) across all samples, accompanied by a noticeable broadening of mesopores (2–50 nm) in THC-1500; meanwhile, the mean pore size of THC hard carbon decreases from 15.53 nm (THC-1300) to 13.07 nm (THC-1500) (Table 1), likely resulting from the merging of adjacent pores during advanced graphitization.

**Figure 3:**
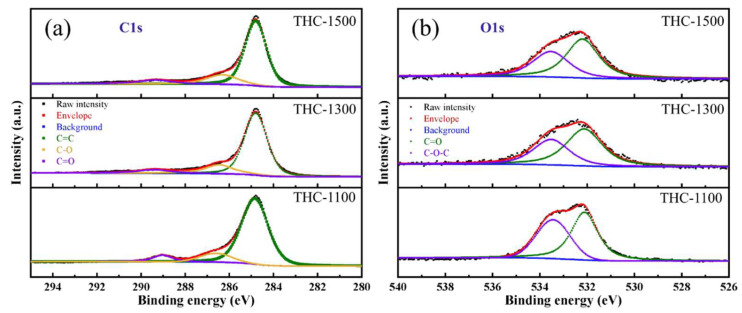
XPS spectra of (**a**) C1s, and (**b**) O1s of THC-1100, THC-1300 and THC-1500.

The FT-IR spectra of THC-1100, THC-1300 and THC-1500 (Figure S3) reveal distinct evolution of surface chemistry and carbon structure with increasing carbonization temperature, providing critical insights into the correlation between synthesis conditions and material properties. Peaks centered at 1100, 1610, 1730 and 3400 cm^−1^ were observed in THC-1100, THC-1300 and THC-1500, which are attributed to the stretching vibrations of C–O–C, C=C, C=O and O–H, respectively. The difference is that an additional absorption peak at 2925 cm^−1^ was detected in THC-1100, assigned to the asymmetric stretching vibration of C–H. This originates from the incomplete carbonization of waste towels. Therefore, temperature is a crucial factor influencing the surface functional groups of hard carbon. X-ray photoelectron spectroscopy (XPS) was employed to investigate the surface chemical states of the waste-towel-derived hard carbons, with a focus on carbon (C1s) and oxygen (O1s) functional groups (Figure 3). These groups play a critical role in surface-driven sodium storage (slope capacity) and electrolyte wettability. C1s spectra (Figure 3a) exhibit three deconvoluted peaks: the dominant peak at ~284.8 eV corresponds to sp^2^-hybridized graphitic carbon (C=C), while peaks at ~286.6 eV and ~289.4 eV are assigned to sp^3^-hybridized aliphatic carbon (C–O)and carbonyl groups (C=O), respectively. With increasing carbonization temperature from 1100 °C to 1500 °C, the intensity of the C=O peaks gradually disappearing. This trend confirms the thermal decomposition of oxygen-containing functional groups and the conversion of sp^3^ defects to sp^2^ graphitic carbon at higher temperatures, consistent with Raman and XRD results showing increased graphitization. The O1s spectra (Figure 3b) further corroborate these changes, revealing three key features: a peak at ~531.2 eV from carbonyl oxygen (C=O), a peak at ~532.3 eV from thermally stable C–O–C bridging groups (epoxy/ether), and a broad peak at ~533.8 eV from physisorbed oxygen or water. With rising temperature, the C=O peak weakens significantly, while the C–O–C peak remains relatively stable, suggesting the preferential decomposition of labile carbonyl groups over more robust ether/epoxy configurations. The overall reduction in oxygen content (evidenced by the diminished intensity of all O1s peaks) aligns with the thermal degradation of the precursor’s cellulose structure, where higher temperatures drive the elimination of oxygen via decarbonylation and dehydration reactions. These chemical state evolutions directly influence the electrochemical performance of the hard carbons. The increasing graphitization (strengthened sp^2^ C=C peak) enhances interlayer Na^+^ insertion (platform capacity), while the reduction in defects and labile oxygen groups (attenuated C–O/C=O peaks) minimizes irreversible side reactions (e.g., electrolyte decomposition), improving initial Coulombic efficiency (ICE).

**Figure 4:**
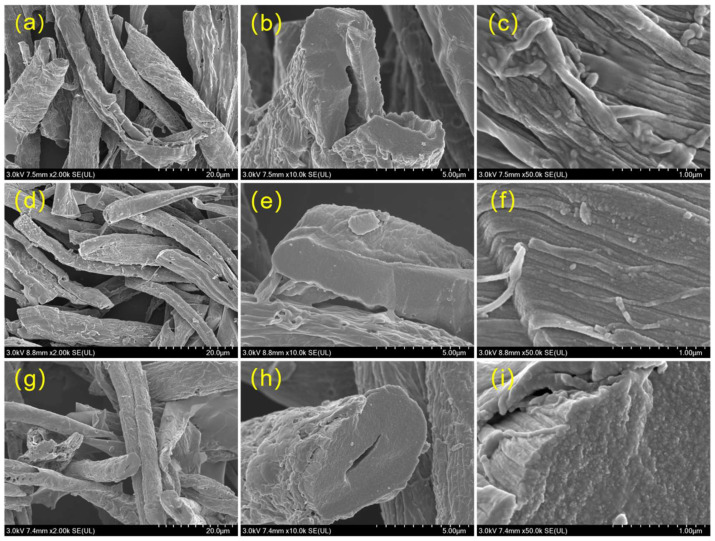
2000× magnification SEM images of (**a**) THC-1100, (**d**) THC-1300 and (**g**) THC-1500; 10,000× magnification SEM images of (**b**) THC-1100, (**e**) THC-1300 and (**h**) THC-1500; 50,000× magnification SEM images of (**c**) THC-1100, (**f**) THC-1300 and (**i**) THC-1500.

The surface morphology and microstructure of hard carbon materials are critical factors influencing their electrochemical performance, as they directly affect ion diffusion pathways, active site accessibility and electrode–electrolyte interface stability. Figure 2 presents scanning electron microscopy (SEM) images of waste-towel-derived hard carbons (THCs) prepared at different carbonization temperatures (1100 °C, 1300 °C and 1500 °C), observed at magnifications of 2000×, 10,000× and 50,000×. The scanning electron microscopy (SEM) images in Figure 4 demonstrate that all waste-towel-derived hard carbons (THC-1100, THC-1300, THC-1500) retain the fibrous morphology inherent to their cellulose precursor, a key structural advantage for sustainable anode design and mechanical stability. At low magnification (Figure 2a,d,g), the THCs exhibit elongated fibrous bundles with lengths of 30–50 μm (Figure S2) and diameters of 5–10 μm, directly replicating the macroscopic dimensions of the original towel fibers. High-magnification cross-sectional views (Figure 2b,e,h) reveal smooth, pore-free surfaces for all samples: an outcome of the pre-oxidation and subsequent acid treatment steps, which effectively removed metal ions and impurities, preventing metal-catalyzed carbon corrosion and avoiding the formation of structural defects. This smooth surface morphology is consistent with the extremely low specific surface areas (~2 m^2^/g) measured via nitrogen adsorption–desorption, confirming the absence of significant porous structures and validating the effectiveness of the purification process in preserving a dense, non-porous carbon skeleton. A striking nanoscale feature emerges at higher magnification (Figure 2c,f,i): individual THC particles are composed of interconnected nanofilaments, a direct replication of the towel’s native fiber bundle structure. This hierarchical nanofilamentary architecture is highly beneficial for electrochemical performance: the continuous network of nanofilaments facilitates the formation of a robust, long-range conductive pathway, enhancing electron transport throughout the electrode and mitigating polarization during high-rate cycling. Meanwhile, the short diffusion distances within the nanofilaments enable rapid sodium-ion (Na^+^) intercalation, a critical prerequisite for high-rate capability. Additionally, the retention of the fibrous morphology from the precursor provides mechanical resilience, buffering volume changes during repeated Na^+^ insertion/extraction cycles and improving long-term cycling stability. Notably, the consistency in morphological features, including fibrous dimensions, smooth cross-sections, and nanofilamentary structure—across all carbonization temperatures (1100–1500 °C) indicates that the pre-treatment and carbonization processes effectively preserve the desirable structural traits of the towel precursor while optimizing the carbon’s graphitic order for Na^+^ storage. This structural fidelity, combined with the low surface area (minimizing irreversible solid electrolyte interface formation) and hierarchical nanofilament network (enhancing transport kinetics), positions these THCs as promising anode materials for sodium-ion batteries, where a balance of capacity, rate capability, and cyclability is essential. The retention of the precursor’s fibrous morphology also underscores the sustainability of this approach, as it leverages waste biomass to create high-value energy storage materials with tailored structural properties.

**Figure 5:**
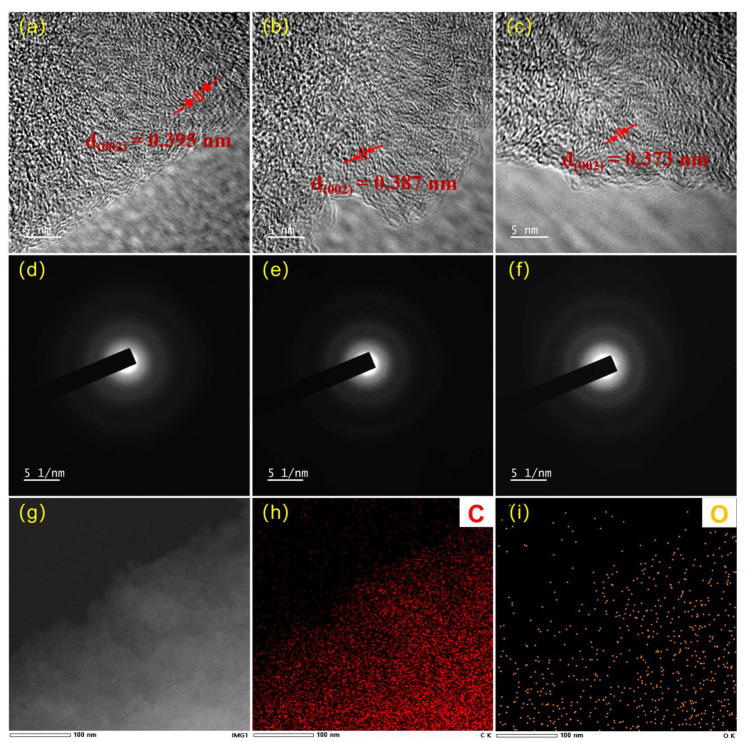
HR-TEM images of (**a**) THC-1100, (**b**) THC-1300 and (**c**) THC-1500; (**d**–**f**) is the corresponding SAED patterns of THC-1100, THC-1300 and THC-1500; EDS elemental mapping of THC-1300; (**g**) TEM morphology image of THC-130; (**h**) C mapping and (**i**) O mapping of THC-1300.

Transmission electron microscopy (TEM) and energy-dispersive X-ray spectroscopy (EDS) provide direct insights into the atomic-scale structure and elemental distribution of the waste-towel-derived hard carbons, complementing the bulk characterizations in Figure 2 and Figure 3. High-resolution TEM (HRTEM) images (Figure 5a–c) reveal the turbostratic nature of the hard carbons, with short-range ordered graphitic layers embedded in an amorphous matrix. For THC-1100 (Figure 5a), the (002) lattice fringes are loosely packed with a measured interlayer spacing d_(002)_ of 0.395 nm, consistent with XRD results. The layers are curved and discontinuous, indicating abundant structural defects-likely a result of incomplete carbonization and residual oxygen functional groups [19]. As the carbonization temperature further increases, the abundance of graphitic-like microcrystals rises, and the interlayer spacing of these graphitic-like layers further decreases to 0.373 nm. All samples display diffuse halos centered at ~2.6 nm^−1^ (corresponding to d_(002)_) and ~4.8 nm^−1^ (corresponding to (100) planes), characteristic of amorphous carbon with short-range order. EDS elemental mapping (Figure 5g–i) visualizes the distribution of carbon (C) and oxygen (O) in THC-1300. The high-angle annular dark-field (HAADF) image (Figure 5g) shows a homogeneous matrix with no obvious phase separation. The C map (Figure 5h) confirms uniform carbon distribution, while the O map (Figure 5i) reveals sparse, isolated oxygen-rich domains (bright yellow spots). These residual oxygen species are likely associated with surface functional groups (e.g., C=O) or trapped in defects, as they do not form large oxide clusters.

**Figure 6:**
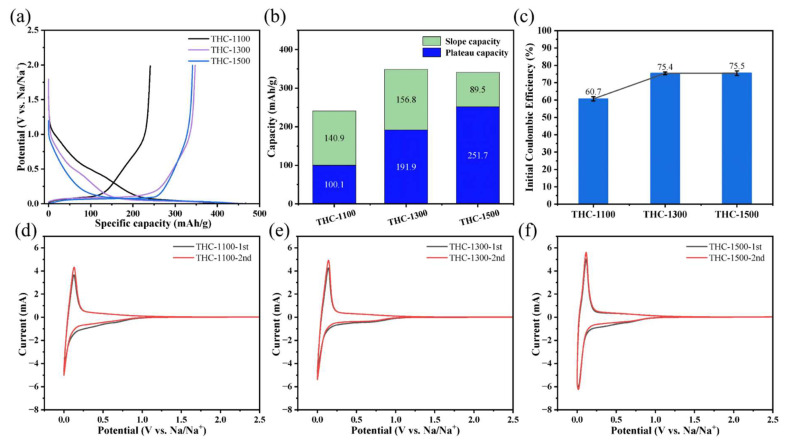
(**a**) First-cycle discharge/charge profiles at 0.1 C (1 C = 320 mA g^−1^) (potential vs. specific capacity), (**b**) Reversible charge capacity decomposition into slope (<0.1 V) and platform (0.1–2.5 V) capacities at 0.1 C, (**c**) Initial Coulombic efficiency (ICE). (**d**–**f**) Cyclic voltammetry (CV) curves of (**d**) THC-1100, (**e**) THC-1300, (**f**) THC-1500 at 0.5 mV s^−1^, showing 1st (dark) and 2nd (red) cycles (0.0–2.5 V vs. Na^+^/Na).

Figure 6a displays the first-cycle galvanostatic charge–discharge (GCD) profiles of THC-1100, THC-1300 and THC-1500 at 0.1 C. All samples exhibit the characteristic two-region behavior of hard carbons: a sloping voltage region (0.1–1.5 V, surface adsorption) and a plateau region (<0.1 V, interlayer Na^+^ insertion). A clear temperature-dependent trend emerges: as carbonization temperature increases from 1100 °C to 1500 °C, the sloping region shrinks progressively, while the plateau region expands, indicating a transition from surface-dominated to interlayer-dominated sodium storage. Figure 6b quantifies the first charge capacity decomposition into slope (surface adsorption) and plateau (interlayer insertion) contributions. With rising temperature, the slope capacity decreases monotonically from 140.9 mAh/g (THC-1100) to 89.5 mAh/g (THC-1500), while the plateau capacity increases sharply from 100.1 mAh/g (THC-1100) to 191.9 mAh/g (THC-1500) before plateauing at 253.7 mAh/g (THC-1500). The total reversible capacity peaks at THC-1300 (348.7 mAh/g), reflecting a balance between slope and plateau mechanisms. Figure 6c presents the initial coulombic efficiency (ICE) of the samples, which improves with carbonization temperature: 60.7% (THC-1100), 75.4% (THC-1300) and 75.5% (THC-1500). As demonstrated by the infrared spectroscopy results, the trend in initial coulombic efficiency originates from the reduction of surface functional groups on the hard carbon, which minimize irreversible electrolyte decomposition and solid electrolyte interface (SEI) formation. Furthermore, the improved ICE and capacity values are comparable to, or even exceed, those reported in previous studies (Table 2). Figure 6d–f present the first- and second-cycle CV curves of THCs at 0.5 mV/s. THC-1300 displays the narrowest high-voltage irreversible peak (0.8–1.2 V, SEI formation/functional group reduction) and the sharpest low-voltage reversible peak (<0.1 V, Na^+^ intercalation) in the first cycle, with nearly identical second-cycle overlap. These traits—rooted in its moderate oxygen content, optimal interlayer spacing (d_(002)_ = 0.387 nm), and balanced defect density (I_D_/I_G_ = 1.07)-minimize irreversible Na^+^ consumption, yielding high initial Coulombic efficiency (~75.4%).

**Figure 7:**
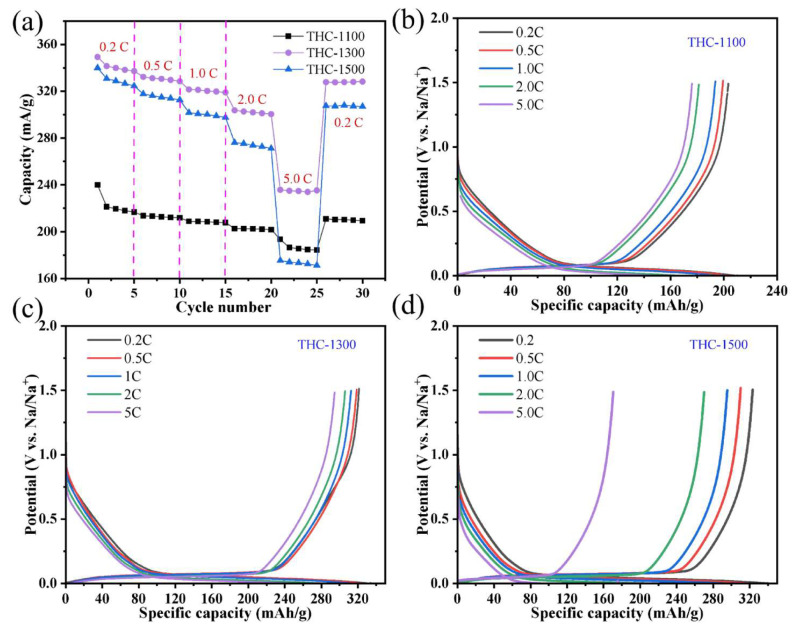
(**a**) Rate capability of THC-1100, THC-1300, and THC-1500 at current densities ranging from 0.2 C to 5.0 C (1 C = 320 mA/g); (**b**–**d**) Galvanostatic charge–discharge profiles of (**b**) THC-1100, (**c**) THC-1300, and (**d**) THC-1500 at various rates (0.2 C to 5 C).

Figure 7a compares the rate capability of THC-1100, THC-1300 and THC-1500 at varying current densities (0.2–5.0 C, where 1 C = 320 mA/g). At low rates (0.2 C), THC-1300 delivers the highest capacity (349.3 mAh/g), followed by THC-1500 (339.5 mAh/g) and THC-1100 (240.0 mAh/g). As the current density increases to 5.0 C, THC-1300 retains the best capacity (234.8 mAh/g, 67.2% of its 0.2 C capacity), while THC-1500 (173.4 mAh/g, 51.0%) show more significant capacity fading. Notably, THC-1300 maintains a stable capacity even after returning to 0.2 C after 30 cycles, indicating excellent rate recoverability. A comparison of the galvanostatic charge–discharge curves for the three materials reveals distinct degradation trends under increasing current rates. For THC-1500, excessive graphitization likely results in narrowed graphite-like interlayer spacing, causing rapid decay of plateau capacity (<0.1 V). Concurrently, diminished surface functional groups accelerate the fading of slope capacity (0.1–1.5 V) (Figure 7d). THC-1100 suffers from fast plateau capacity degradation due to insufficient graphitization and poor electronic conductivity, though its retained functional groups mitigate slope capacity decay (Figure 7b). In contrast, THC-1300 exhibits optimal performance (Figure 7c): its engineered graphite-like interlayer spacing ensures high-rate stability of plateau capacity, while moderate defect density minimizes slope capacity degradation, collectively delivering superior comprehensive properties.

**Figure 8:**
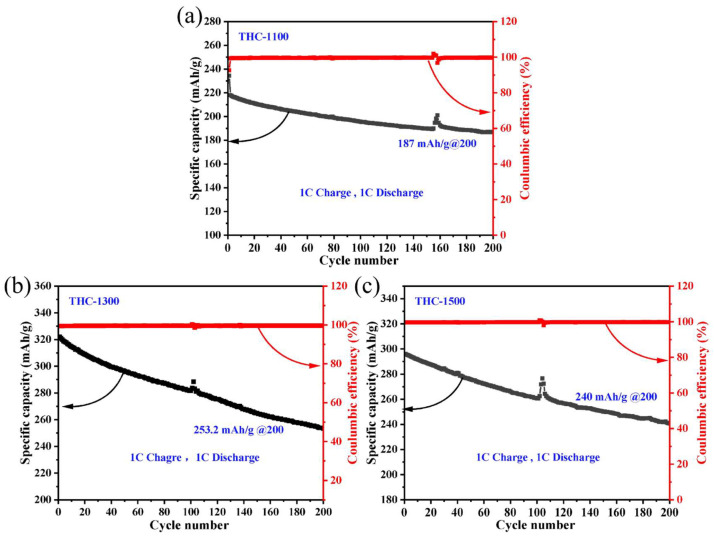
Long-term cycling performance and Coulombic efficiency of biomass-derived hard carbons at 1 C. (**a**) THC-1100, (**b**) THC-1300, and (**c**) THC-1500 were charged and discharged at a current density of 1 C (1 C = 320 mA g^−1^).

Figure 8 depicts the long-term cycling stability and Coulombic efficiency (CE) of THC-1100, THC-1300, and THC-1500 over 200 cycles at 1 C (320 mA g^−1^). After 200 cycles, THC-1100 retains a discharge capacity of 187 mAh g^−1^ (81% retention), while THC-1300 and THC-1500 deliver 253.2 mAh g^−1^ (78.8% retention) and 240 mAh g^−1^ (79.1% retention), respectively. The CE stabilizes at 99.6% throughout cycling, demonstrating exceptional electrochemical reversibility. Notably, this high-rate cycling performance (1 C) confirms the superior fast-charging capability of THC hard carbons. The outstanding stability originates from: (1) low specific surface area (<3 m^2^/g minimizing electrolyte decomposition; (2) formation of a robust solid electrolyte interphase (SEI) during initial cycles that effectively suppresses continuous side reactions and irreversible Na^+^ consumption; (3) Optimized graphite-like interlayer spacing (~0.38 nm), ensuring reversible sodium-ion intercalation/deintercalation. These characteristics establish THC hard carbons as promising anode candidates for sodium-ion batteries.

Based on the above results, we aim to elucidate the sodium storage mechanism of the composite THC hard carbon. As mentioned previously, infrared spectroscopy results demonstrate that as the carbonization temperature increases from 1100 °C to 1500 °C, the surface functional groups of THC gradually decrease. Correspondingly, the initial coulombic efficiency (ICE) of the HC hard carbon gradually increases, while the slope capacity shows a declining trend. This suggests that the slope capacity originates from surface adsorption. Furthermore, with increasing temperature, graphitic-like microcrystals become more abundant. As heteroatoms are further removed, samples obtained at higher temperatures exhibit a higher degree of defects in Raman spectroscopy results. In line with this, THC-1500 possesses the highest plateau capacity. Therefore, the plateau capacity is more likely attributable to sodium ion intercalation. In addition, THC-1300 and THC-1500 exhibit similar total capacities during the first discharge. The difference in their plateau and slope capacities mainly stems from a mutual conversion of capacity contributions. During rate performance tests, THC-1500 consistently shows a lower capacity retention rate than THC-1300, with this difference being most pronounced at a 5 C discharge rate. For THC-1500, the plateau capacity accounts for 73.7% of the total capacity, and the capacity fading at 5 C primarily originates from the decay of the plateau capacity. This decay ultimately results from a smaller interlayer spacing, which is unfavorable for the rapid intercalation of sodium ions. Moreover, all THC samples possess a very small specific surface area, and the contribution from pore-filling is minimal. Consequently, the sodium storage mechanism of THC aligns more closely with the “adsorption-intercalation” model.

## 4. Conclusions

In summary, this work demonstrates that waste-towel-derived hard carbon anodes with nanocarbon fiber bundle microstructures, optimized through controlled carbonization at 1300 °C, achieve superior sodium storage performance by balancing critical structural parameters: an ideal interlayer spacing (0.387 nm), low defect density (I_D_/I_G_ = 1.07), and low surface area (1.62 m^2^/g). Scientifically, we reveal that this unique combination enables simultaneous enhancement of ionic diffusion kinetics and defect-mediated adsorption, yielding exceptional rate capability (67.2% capacity retention at 5 C) and cycling stability (78.8% retention after 200 cycles at 1 C), surpassing most reported biomass-derived hard carbons. the sodium storage process in THC hard carbon conforms to the “adsorption-intercalation” model. Practically, Through a pre-oxidation/acid-washing/carbonization strategy, this work establishing a sustainable “waste-to-energy” paradigm for anode manufacturing. These findings provide both fundamental insights into structure–property relationships in hard carbons and a scalable approach to eco-friendly battery materials development.

## Figures and Tables

**Table 1:** Structural and spectroscopic parameters of hard carbon prepared at different temperatures.

Sample ID	d(002) (nm)	Raman (I_D_/I_G_)	BET Surface Area (m^2^/g)	BJH Desorption Pore Volume (cm^3^/g)	Average Pore Diameter (nm)	Median Micropore Diameter (nm)
THC-1100	0.395	1.01	1.44	0.0057	15.53	1.190
THC-1300	0.387	1.07	1.62	0.0059	14.25	1.072
THC-1500	0.373	1.11	2.22	0.0073	13.07	1.076

**Table 2:** Electrochemical performance parameters of biomass derived hard carbon.

Biomass Source	ICE (%)	1st Charge Capacity (mAh/g)	Current Density (mA/g)
Garlic peel [27]	41	258	100
Bagasse [21]	-	307	30
Cellulose [28]	84	376	30
Peanut shells [29]	61	210	20
Rice husk [30]	34	136	30
This work	74.5	348.7	32

## Data Availability

The original contributions presented in this study are included in the article/Supplementary Materials. Further inquiries can be directed to the corresponding author.

## Supplementary Materials

The following supporting information can be downloaded at: https://www.mdpi.com/article/10.3390/polym18020206/s1, Figure S1: Yield of different THCs; Figure S2: 500× magnification SEM images of (a) THC-1100, (b) THC-1300 and (c) THC-1500; Figure S3: FT-IR spectra of THC samples carbonized at different temperatures (1100 °C, 1300 °C, and 1500 °C).
