# Supplementary material for: Waste-Towel-Derived Hard Carbon as High Performance Anode for Sodium Ion Battery
Source: Polymers (Basel). 2026 Jan 12;18(2):206. doi: 10.3390/polym18020206 (PMC12845708; doi:10.3390/polym18020206)
Supplement: Supplementary file 1 [file polymers-18-00206-s001.zip › polymers-4054909-supplementary.pdf]

Waste-Towel-derived Hard Carbon as High Performance Anode for Sodium Ion Battery

Daofa Ying[a*], Kuo Chen[a], Jiarui Liu[a], Ziqian Xiang[a], Jiazheng Lu[a], Chuanping Wu[a], Baohui Chen[a], Yang Lyu[a], Yutao Liu[a], Zhen Fang[a]

[a] State Key Laboratory of Disaster Prevention and Reduction for Power Grid Transmission and Distribution Equipment, State Grid Hunan Electric Company Limited Disaster Prevention and Reduction Center, Changsha, Hunan, P.R. China.

* To whom correspondence should be addressed.

Tel.: +86-0731-86332056.

E-mail: 17364041396@163.com (D. Ying).

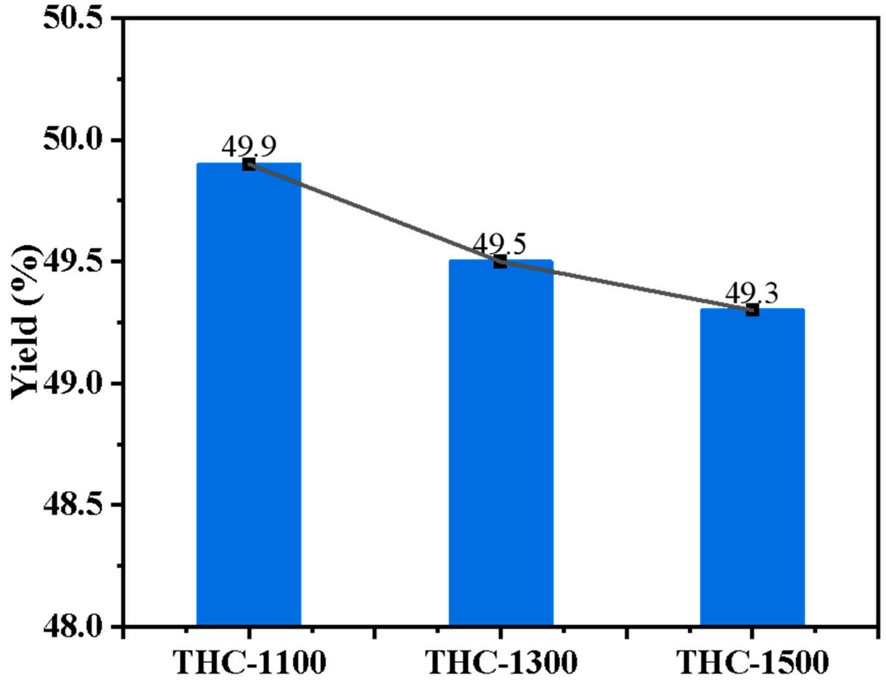

Figure S1. Yield of different THCs.

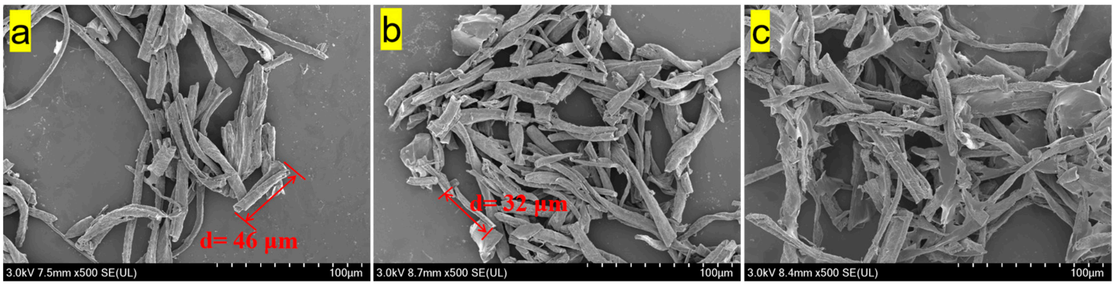

Figure S2. 500× magnification SEM images of (a) THC-1100, (b) THC-1300 and (c) THC-1500.

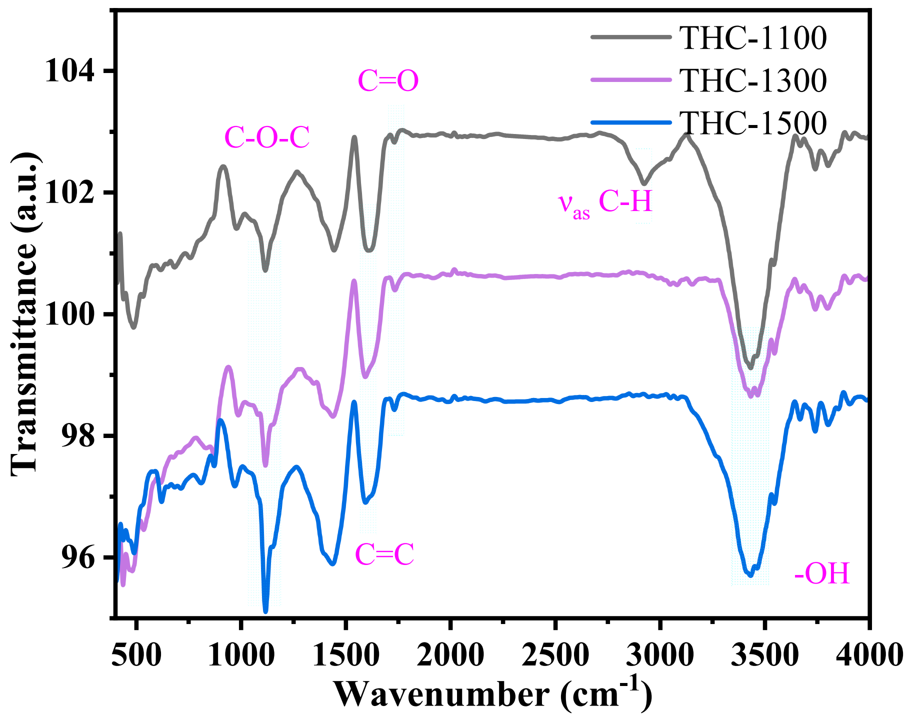

Figure S3. FT-IR spectra of THC samples carbonized at different temperatures (1100℃, 1300℃, and 1500℃).
